# Evolutionary ingestive-digestive homeostatic dysregulation theory

**DOI:** 10.1515/jtim-2026-0064

**Published:** 2026-07-21

**Authors:** Enqiang Linghu

**Affiliations:** Department of Gastroenterology, the First Medical Center of Chinese PLA General Hospital, Beijing, China

## Introduction

Modern digestive, metabolic, immune, and neoplastic diseases may partly arise from a sustained decoupling between modern ingestive patterns and the evolutionarily shaped “physiological programs” of the human organism and the modern social environment. This decoupling is particularly evident in the relationship between digestive intake and the intrinsic physiological configuration of the body. On this basis, Professor Enqiang Linghu independently conceptualized and proposed the Evolutionary Ingestive–Digestive Homeostatic Dysregulation Theory (hereinafter referred to as the Theory), which argues that modern lifestyles have substantially deviated from the adaptive state formed during long-term human evolution. The Theory offers a conceptual framework for understanding, preventing, and intervening in digestive diseases, tumorigenesis, immune dysregulation, metabolic disorders, and related systemic diseases (Figure 1).

**Figure 1:**
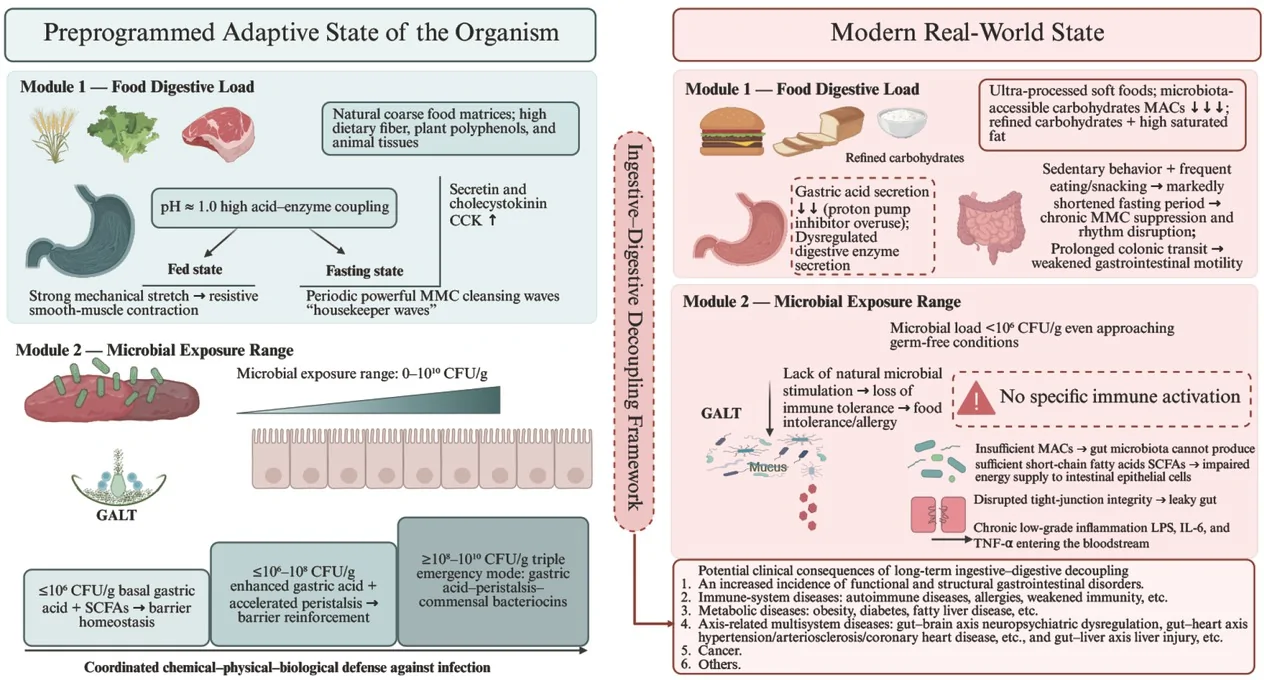
Conceptual framework of the Evolutionary Ingestive–Digestive Homeostatic Dysregulation Theory: the Ingestive–Digestive Decoupling Framework. CCK: cholecystokinin; CFU: colony-forming unit; GALT: gut-associated lymphoid tissue; MAC: microbiota-accessible carbohydrate; MMC: migrating motor complex; SCFA: short-chain fatty acid; LPS: lipopolysaccharide; IL-6: interleukin-6; TNF-α: tumor necrosis factor-alpha.

## Core definition of the theory

The Theory proposes that the human digestive system evolved to adapt to specific food structures, microecological exposures, eating rhythms, physical activity patterns, and environmental stressors. Chronic exposure to the modern digestive exposome—including ultra-processed foods, insufficient dietary fiber and microbiota-accessible carbohydrates (MACs), frequent eating, circadian disruption, sedentary behavior, excessive sanitization, chronic psychosocial stress, and pharmacological acid suppression—may induce multilayered digestive homeostatic dysregulation, particularly in genetically susceptible individuals or those with sustained high exposure.^[1, 2, 3, 4]^

This dysregulation involves a mutually reinforcing network across six regulatory domains: the gut microbiota, gastrointestinal motility, the mucosal barrier, the enteroendocrine system, the gastric acid barrier, and nutrient absorption. These modules correspond to ecological input, motility-mediated clearance, interface defense, signal sensing, chemical barrier function, and nutritional output, together covering the essential sequence of “intake-processing-barrier-signaling-absorption”. Their interactions may further influence the microecology-gut-brain axis, the gut-liver-immunometabolic axis, and systemic low-grade inflammation.^[5, 6, 7]^ Because most modules have measurable indicators, the Theory can be tested in cohort studies, mechanistic experiments, and intervention trials.

## Decoupling between the modern exposome and evolutionary physiological programs

From an evolutionary perspective, the digestive system was preprogrammed for natural, coarse food matrices rich in fiber and polyphenols, including resistant starch, plant polyphenols, natural proteins, and minimally processed animal and plant tissues.^[1,3]^ Such foods provide energy, mechanical stretch, and biochemical stimulation. Their texture promotes resistive smooth-muscle contraction, gastric distension, acid and enzyme secretion, bile excretion, enteroendocrine responses, and distal microbial fermentation. During fasting, the migrating motor complex (MMC) provides periodic cleansing, forming a biphasic motility pattern of load-dependent processing and intermittent clearance.

The gastrointestinal tract is also not designed to function as a sterile system. Barrier homeostasis depends on gastric acid, bile, mucus, peristalsis, gut-associated lymphoid tissue (GALT), and commensal microbiota. The evolutionarily preprogrammed state likely involved repeated exposure to food-associated microorganisms from the natural environment. Such exposure contributes to immune education and tolerance. In this framework, low microbial loads are managed by basal acid secretion and short-chain fatty acids (SCFAs), moderate loads trigger stronger acid secretion and peristalsis, and high loads may activate a coordinated “gastric acid-peristalsis-bacteriocin” defense response.

Modern lifestyles often move in the opposite direction. Highly sanitized foods may reduce natural microbial stimulation, leaving the immune system relatively under-stimulated yet prone to sensitization, thereby increasing susceptibility to food intolerance or allergy. Ultra-processed foods are typically soft, energy-dense, highly palatable, and low in fiber, resistant starch, polyphenols, and MACs.^[2,4]^ Persistent MAC deficiency may reduce microbial SCFA production, impair epithelial energy supply and tight-junction integrity, and promote mucus degradation, intestinal permeability, and chronic low-grade inflammation.^[4, 5, 6]^ Sedentary behavior, frequent meals, nocturnal eating, sleep deprivation, and circadian disruption may further weaken MMC-mediated cleansing and disturb host metabolism and microbiota rhythms.^[8]^

Gastric acid-enzyme coupling and neuroendocrine regulation are also central to the Theory. Gastric acid supports protein digestion and limits bacterial overcolonization of the upper gastrointestinal tract. Long-term or inappropriate acid suppression, chronic stress, and reduced vagal tone may alter acid secretion, motility, and mucosal repair. Imhann *et al*.^[9]^ provide indirect support that proton pump inhibitor-associated hypochlorhydria can alter the gastrointestinal microbiome and may increase susceptibility to small intestinal bacterial overgrowth (SIBO).

## Core pathophysiological network of the theory

The pathophysiology of the Theory should not be viewed as a linear causal chain, but as a feedback network involving six interdependent modules. Chronic disturbance of one component may influence the others, although dominant drivers and clinical phenotypes may vary among individuals.

Low-fiber, low-MAC, and high-ultra-processed diets may reduce gut microbial diversity and alter SCFA production.^[3, 4, 5]^ SCFAs, especially butyrate, provide energy to colonic epithelial cells and participate in immune regulation, barrier maintenance, and enteric nervous system signaling. Conversely, abnormal motility may cause luminal retention, alter redox state, bile acid exposure, and fermentation conditions, and thereby reshape the microbiota. Thus, the gut microbiota and motility likely form a bidirectional cycle.

The mucosal barrier is the key interface separating nutrients, commensals, and potential pathogens. Altered microbial metabolism, mucus thinning, tight-junction dysfunction, and local inflammation may increase intestinal permeability and facilitate contact between microbial-associated molecular patterns and the mucosal immune system.^[6]^

Enteroendocrine cells sense nutrients, microbial metabolites, and neural signals. Changes in food structure or microbial metabolism may affect 5-hydroxytryptamine (5-HT), glucagon-like peptide-1 (GLP-1), peptide YY (PYY), cholecystokinin (CCK), and motilin, thereby regulating gastric emptying, peristalsis, appetite, insulin secretion, and visceral sensitivity. Consistent with this view, Feng *et al*. reviewed evidence that gut microbiota may modulate postprandial glucose responses through microbial metabolites such as short-chain fatty acids, bile acids, and trimethylamine N-oxide, thereby linking digestive microecology to metabolic homeostasis^[10]^. When gastric acid defense and enteroendocrine signaling are simultaneously disturbed, abnormal gastric emptying, SIBO tendency, and persistent upper abdominal symptoms may be promoted. Brain-gut-microbiota research further suggests bidirectional feedback among digestive signals, stress, emotion, and visceral sensitivity.^[7]^

Nutrient malabsorption may be a shared downstream consequence. Persistent low-grade inflammation, reduced brush-border function, abnormal bile acid circulation, insufficient acid secretion, and altered transit time may impair the release or absorption of iron, vitamin B12, folate, vitamin D, fat-soluble vitamins, and essential amino acids. Long-term nutritional insufficiency may weaken mucosal repair, immune tolerance, and neurotransmitter synthesis, forming a “nutrition-inflammation-barrier” cycle. This may help explain why digestive symptoms often coexist with fatigue, anxiety, metabolic abnormalities, or immune susceptibility.

## Conclusions and future research directions

The Theory re-examines modern digestive dysfunction and multisystem comorbidities through evolutionary medicine and systems biology. The modern exposome may disturb digestive homeostasis through changes in food matrix, microbial input, eating rhythm, motility, barrier function, enteroendocrine signaling, gastric acid secretion, and nutrient absorption.

Future studies should move beyond cross-sectional disease-control comparisons. Key directions include chronic low-grade inflammation, barrier dysfunction, immune tolerance and allergy, metabolic diseases, gut-brain/gut-liver/gut-heart/gut-reproductive axes, constitutional resilience and chronic fatigue, genomic stability, epigenetic remodeling, penetrance of genetic diseases, tumor susceptibility, and health gradients among populations with different degrees of modernization.
